# Analysis of Toxic Effects of Fluoride on Ovine Follicular Granulosa Cells Using RNA-Seq

**DOI:** 10.3390/antiox13050506

**Published:** 2024-04-24

**Authors:** Tian Ma, Wanruo Liu, Didi Jiang, Guolin Zhang, Xingxu Zhao, Yong Zhang, Zongshuai Li

**Affiliations:** 1 College of Veterinary Medicine, Gansu Agricultural University, Lanzhou 730070, China; mt13893610637@163.com (T.M.); lwr182311@163.com (W.L.); m13830234350@163.com (D.J.); 15293882764@163.com (G.Z.); zhaoxx@gsau.edu.cn (X.Z.); 2 Key Laboratory of Education, College of Pastoral Agriculture Science and Technology, Lanzhou University, Lanzhou 730020, China; 3 State Key Laboratory of Grassland Agro Ecosystems, Lanzhou University, Lanzhou 730020, China; 4 Key Laboratory of Grassland Livestock Industry Innovation, Lanzhou University, Lanzhou 730020, China; 5 Key Laboratory of Agriculture and Rural Affairs, Grassland Agriculture Engineering Center, Lanzhou University, Lanzhou 730020, China

**Keywords:** fluoride, ovine, granulosa cells, RNA-Seq, endoplasmic reticulum stress

## Abstract

Fluoride is abundant in the environment and is an essential trace element in living organisms. However, prolonged excessive fluoride intake can lead to fluorosis, which poses a threat to the reproductive health of animals and humans. Although previous research has mainly focused on animal models, the impact of fluoride on ovine follicular granulosa cells (GCs) has not been comprehensively elucidated. This study employed RNA-Seq technology to elucidate the toxic effects of fluoride on ovine follicular GCs and its mechanism of action. Culturing primary ovine follicular GCs in vitro and subjecting them to fluoride treatment revealed 3218 differentially expressed genes (DEGs), with 2278 upregulated and 940 downregulated. Significantly, this study unveiled fluoride’s induction of endoplasmic reticulum (ER) stress in cells, triggering a cascade involving the PERK pathway factor *ATF4*, leading to cell death via *DDIT3*/*CHOP* activation and the subsequent upregulation of *CHAC1*, *ATF3*, *ERO1α*, and *TRIB3*. These findings provide crucial insights into the toxicity of fluoride in ovine, offering a foundation for mitigating fluoride-related losses in the farming industry.

## 1. Introduction

Fluoride is a nonmetallic chemical element that is naturally abundant, has active chemical properties, and generally exists in the form of compounds [1]. Fluoride has a dual impact on human health: in moderate amounts, it serves as a beneficial trace element for the human body, while excessive long-term intake can lead to fluorosis [2]. Being an accumulative poison, fluoride can be absorbed by plants and pastures. Consumption by cattle and ovine can result in joint enlargement, osteoporosis, and even paralysis. Excessive intake of fluoride destroys the balance between calcium and phosphorus metabolism, resulting in fluorosis, with symptoms such as tooth staining and joint deformation. Various species are susceptible to harm from high fluoride concentrations, with ovine in fluoride-rich areas being particularly sensitive. The primary sources of exposure include drinking water, industrial wastewater, dust, and fluoride-laden feed supplements. Water and roughage are considered primary contributors to ovine fluorosis, resulting in damage to the teeth, bones, and other organs [3].

Low doses of fluoride are beneficial for bone and tooth development [4]. However, prolonged exposure to high fluoride levels can interfere with bone formation and adversely affect bones and teeth, resulting in osteofluorosis and dental fluorosis [5]. Fluoride has a dose-dependent effect on osteoblast proliferation, with low doses of fluoride promoting osteoblast proliferation and high doses inhibiting osteoblast proliferation and promoting apoptosis [6]. Moreover, fluoride exposure is associated with liver and kidney dysfunction [7], changes in intellectual development, and thyroid function in children [8,9]. Numerous studies have demonstrated that excessive intake of fluoride can cause neurotoxicity, leading to learning, memory, and cognitive deficiencies [10], anxiety, and depression-like behaviors [11,12]. Yan et al. explored the role of the toll-like receptor signaling pathway in fluoride-induced cardiotoxicity via high-throughput transcriptome sequencing of rat heart tissues. They confirmed that the toll-like receptor signaling pathway and inflammatory response were inhibited in rat hearts under the effect of sodium fluoride (NaF), thus revealing the mechanism of cardiac fluorosis [13].

Excessive fluoride intake can adversely affect the reproductive system of both females and males [14]. Yang et al. conducted an in vitro study on the toxic effects of fluoride on rat support cells. The results showed that fluoride-exposed supporting cells showed decreased viability, ER stress, and decreased mitochondrial membrane potential levels [15]. NaF induced ovarian apoptosis and caused oxidative stress in rats exposed to fluoride for 6 months. Further studies on GCs showed that NaF activated ERK and JNK signaling pathways, while p38 and PI3K remained unchanged. It was suggested that NaF could cause ovarian dysfunction by inducing apoptosis [16]. A rat model was used to simulate fluoride intake in humans. Fluoride increased MDA accumulation, decreased SOD activity, and enhanced germ cell apoptosis in rats exposed to 25, 50, and 100 mg/L NaF. ER stress was also activated, with elevated mRNA and protein expression levels of GRP78, IRE1 and CHOP. This suggests that, in addition to oxidative stress, ER stress and inflammation are important mechanisms contributing to fluoride-induced spermatogenic disorders and germ cell damage [17]. Chronic fluorosis increases the transcriptional levels of *IL-17*, *IL-6*, and *TNF-α* and the translational levels of IL-17 and TNF-α in the testes of mice, causing an inflammatory response in the testes [18]. Several preclinical studies have shown that fluoride exposure decreases sperm viability [19,20], reduces sperm concentration, and affects sperm capacitation [21,22]. Exposure to high concentrations of fluoride was found to cause ovarian damage in mice [23], and the effects on female mice mainly manifested as a reduction in the pregnancy rate and litter size. Ultrastructural observations of fluoride-exposed uterine tissues showed nuclear ambiguity, a decrease in microvilli, an increase in lysosomes, an expansion of the ER, and vacuolization of the mitochondria [24]. Animal studies have shown that exposure to fluoride reduces the levels of sex hormones, such as testosterone, estradiol, follicle stimulating hormone, and luteinizing hormone [25].

GCs are important components of mammalian follicular structures that coexist in the same microenvironment as oocytes and interact with each other to regulate oocyte growth and maturation [26]. GCs promote oocyte development by providing essential nutrients to the oocyte and are involved in the maintenance of oocyte meiotic inhibition, the induction of oocyte meiosis, and cytoplasmic maturation [27]. Functional collaboration between oocytes and their surrounding GCs is necessary for oocyte growth and the acquisition of developmental competence. Follicles and their developmental processes are closely related to GCs. Therefore, GCs are an ideal cellular model for the study of cell proliferation, differentiation, and signaling [28]. Moreover, the in vitro follicular GC culture system can be used as an evaluation criterion to study the reproductive toxicity of toxins.

Ovine are mammals of the genus *Ovine* in the order Artiodactyla [29]. Ovine, originally from Central Asia, have proliferated globally because of regional environmental variations and evolving social demands. Over many years of selective breeding, the sheep population has become vast, widely dispersed, and diverse in species [30]. Ovine are prevalent in numerous fluoride-affected regions worldwide, sparking increased attention to fluoride because of their growing economic importance [3]. For example, in India and Turkey, which have rich and diverse ovine germplasm resources, fluorosis continues to be a major obstacle for the ovine industry [31,32].

In Africa and Asia, endemic fluorosis accounts for most cases, affecting a very large number of people. Countries located within the fluoride belt, including Egypt, Libya, Turkey, India, China, and Japan, are particularly affected. Recent studies have shown that fluoride induces apoptosis by inducing oxidative stress, modulating intracellular lipid peroxidation, and altering gene expression. Sources of fluoride exposure, encompassing food (such as tea, milk, and meat), medications, water, air, and excessive use of toothpaste, vary; however, drinking water is the primary source of exposure. Fluoride has been found to have toxic effects on mice [24,33], *Xenopus laevis* [34], laying hens [25], pigs [35], and other animals. However, more in-depth research is required to elucidate the molecular mechanisms underlying fluoride toxicity in ovine reproduction, particularly in ovine GCs.

In this study, we investigated the toxic effects of fluoride on ovine GCs and elucidated the possible mechanisms underlying these effects using RNA-Seq technology. The results of this study are expected to facilitate our subsequent screening of fluoride antagonist drugs and ultimately provide a database and theoretical basis for improving the reproductive performance of animals and the quality of livestock products.

## 2. Materials and Methods

### 2.1. Ovary Collection

Ovine ovaries (*n* = 20) were collected from a slaughterhouse in Lanzhou, Gansu Province. Upon ovine slaughter (*n* = 10; body weight = 25–30 kg), the ovaries were promptly collected by clipping with sterile scissors, rinsed with saline solution, and then preserved in PBS (Biosharp, Beijing, China) containing penicillin and streptomycin at 37 °C. They were transported back to our laboratory within 2 h of sampling.

### 2.2. In Vitro Cultivation of Primary GCs

The collected ovaries were rinsed with 75% alcohol, excess tissue on the ovary was removed using a sterilized scalpel or surgical scissors, and follicular fluid was collected using a 5 mL syringe (the syringe was changed diligently or sterilized on an alcohol lamp to avoid contamination). The cells were centrifuged at 106 g rpm for 5 min then washed with PBS. The above centrifugation steps were repeated for 5 min, and the cells were finally added to DMEM/F12 culture medium (BI, Kibbutz Beit Haemek, Israel) containing 10% fetal bovine serum (BSA, BI, Kibbutz Beit Haemek, Israel), 1% penicillin, and streptomycin. Gentle agitation was applied to maintain cell suspension and ensure even dispersion of precipitates. The granulocytes were inoculated into cell culture flasks and cultured at 37 °C and 5% CO_2_. After 24 h, the cell growth state was stabilized, and the culture medium was changed. When the cell density reached 70–80%, the cells were digested with trypsin and passaged. The experimental group cells were treated with NaF (Sigma-Aldrich, St. Louis, MO, USA).

### 2.3. Cellular Identification and Cell Viability Assay

The cells were washed with PBS 2–3 times, fixed with 500 µL 4% paraformaldehyde for 30 min, and then incubated with 500 µL of 0.1% Triton for 20 min at room temperature. The cells were washed again with PBS 2–3 times, blocked with 5% BSA for 30 min, and incubated with 300 µL of FSHR antibody (1:300 dilution; BIOSS, Beijing, China) overnight at 4 °C. The next day, we added the secondary antibody at 37 °C for 2 h. The results were visualized by fluorescence microscopy.

After adjusting the cell density to 1 × 10^6^, the cells were inoculated into 96-well plates (Corning, NY, USA), with 100 µL of cell suspension per well, and placed at 37 °C under 5% CO_2_ for preculturing. The experimental groups were set as the control (CK) and experimental (T1, T2, T3, T4, T5, T6, and T7, with NaF concentrations of 2, 2.5, 3, 3.5, 4, 4.5, and 5 mM, respectively). After pre-incubation, 10 µL of CCK solution (Invigentech, Irvine, CA, USA) was added to each well, with care taken to avoid the formation of air bubbles in the wells, which could affect the OD readings. The plates were incubated in an incubator for 4h. The absorbance at 450 nm was measured using an enzyme meter (Molecular Devices, Shanghai, China).

### 2.4. Screening of NaF Working Concentration

Based on the results of the CCK8 experiment, the experimental groups were set as the control (CK) and experimental (T1, T2, and T3, with NaF concentrations of 3.5, 4, and 4.5 mM, respectively). The cells were resuspended, and the density was adjusted to inoculate into 6-well plates (Corning, NY, USA). After 24 h, 350 µL of TRIzol (Thermo Fisher Scientific, Waltham, MA, USA) was added to each well, and the cells were gently lysed. Cells were collected into sterile, enzyme-free 1.5 mL centrifuge tubes. The total cellular RNA was extracted and stored at −80 °C. The RNA was reverse-transcribed to cDNA according to the manufacturer’s instructions (Thermo Fisher Scientific, Waltham, MA, USA), 2 × M5 HiPer SYBR Premix Es Taq (Mei5bio, Beijing, China) was used for qRT-PCR. Considering the studies indicating fluoride’s potential to induce cellular toxicity through oxidative stress, apoptosis, and autophagy, we selected specific genes for investigation. These included the apoptosis-related genes *CASP3*, *CASP8,* and *P53*; oxidative stress-related genes *SOD1*, *SOD2*, *CAT*, and *GPX1*; and autophagy-related genes *LC3*.

### 2.5. RNA Extraction, Library Construction, and Sequencing

cDNA library sequencing was performed using an Illumina HiSeqTM 2500/4000 (Gene Denovo Biotechnology Co., Ltd., Guangzhou, China). Bioinformatics analysis was performed using OmicSmart, a real-time interactive online platform for data analysis (http://www.omicsmart.com (accessed on 7 March 2024)).

### 2.6. Immunofluorescence Detection of Cellular ROS Levels

The reactive oxygen species (ROS) levels in the GCs were assayed according to the instructions of the ROS Assay Kit (BioVision, Milpitas, CA, USA). At the end of cell incubation in 96-well plates, 100 µL of the ROS assay buffer was slowly added after discarding the medium for 5 min. The supernatant was then discarded, and the cells were rinsed slowly once with PBS. Subsequently, 100 μL of 1 × ROS labeling buffer was added, and the cells were left to incubate at 37 °C for 45 min, avoiding light. The supernatant was discarded again, 100 μL of ROS inducer buffer was added, and the mixture was transferred at 37 °C for 1 h under light protection. The cells were then observed under an inverted fluorescence microscope.

### 2.7. Analysis of RNA-Seq Data and qRT-PCR

To validate the accuracy of RNA-Seq, nine genes (*PIK3CA*, *ATF4*, *DDIT3*, *SIRT1*, *VEGFA*, *SLC1A5*, *GDF15*, *P53,* and *BAX*) were randomly selected for qRT-PCR analysis. The total RNA was extracted after cell collection, and qRT-PCR was performed for verification after the reverse transcription of RNA into cDNA, according to the manufacturer’s instructions (Thermo Fisher Scientific, Waltham, MA, USA). Then, 2×M5 HiPer SYBR Premix Es Taq (Mei5bio, Beijing, China) was used for and subjected to qRT-PCR. The amplification program was set up using a two-step method with 45 cycles, and each group of samples was analyzed 3 times. After validation of the RNA-Seq results through qRT-PCR, the enriched Gene Ontology (GO) terms and Kyoto Encyclopedia of Genomes (KEGG) pathways were histologically analyzed and supplemented with genome enrichment analysis (GSEA) to screen out genes and related signaling pathways of significance, and to make a preliminary inference on the mechanism of fluoride action on ovine follicular GCs. Protein–protein interaction (PPI) was constructed by a string online database (https://cn.string-db.org/ (accessed on 19 March 2024)) and Cytoscape software (version 3.3.0). The primer sequences are presented in Table 1.

### 2.8. Data Analysis

All experiments were subjected to three or more technical replications, and the obtained data were analyzed using GraphPad statistical software (version 9.0). The results were expressed as the mean ± SD. For the qRT-PCR and ROS results, differences between the two groups were analyzed by Student’s *t*-test. One-way analysis of variance, followed by Dunnett’s multiple comparisons test, was used to compare the groups’ CCK8 results. *p* < 0.01 (**) indicates a highly significant difference, and *p* < 0.05 (*) indicates a significant difference.

## 3. Results

### 3.1. Cellular Identification and Cell Viability Assay

**Figure 1:**
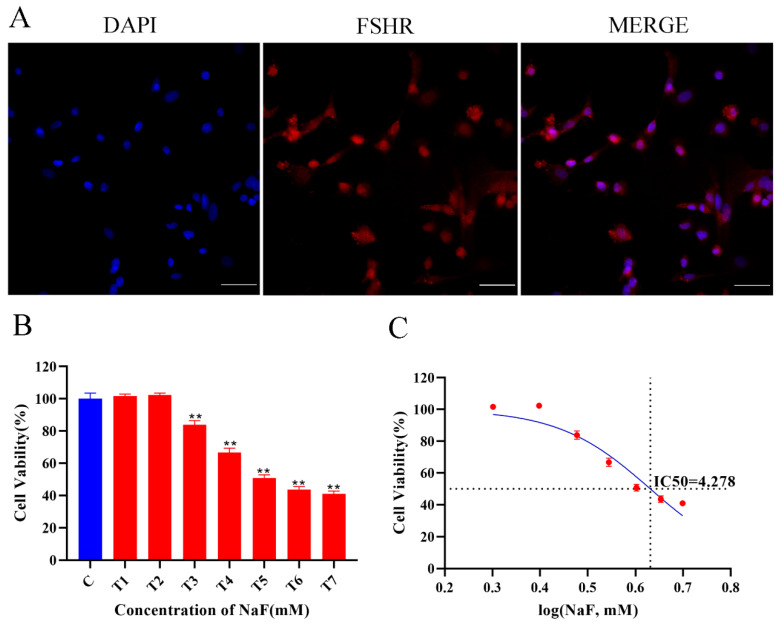
(**A**) Identification of ovine follicular GCs (10×, 100 µm). (**B**) Effects of NaF on cell viability of ovine GCs. All experiments were repeated at least 3 times. ** *p* < 0.01. (**C**) IC50 curve after log conversion.

The isolated and cultured ovine follicular GCs were identified using immunofluorescence. The immunofluorescence results show that the isolated and cultured primary cells were ovine follicular GCs (Figure 1A). The effect of fluoride on ovine GCs was determined using a CCK-8 kit. Figure 1B shows that cell viability was significantly reduced with an increasing NaF dose in a dose-dependent manner. The IC_50_ of NaF in the cells was calculated to be 4.278 mM. Therefore, 0, 3.5, 4, and 4.5 mM were initially set as the NaF working concentrations to screen for the final working concentration of NaF in subsequent experiments.

### 3.2. NaF Final Concentration Screening

**Figure 2:**
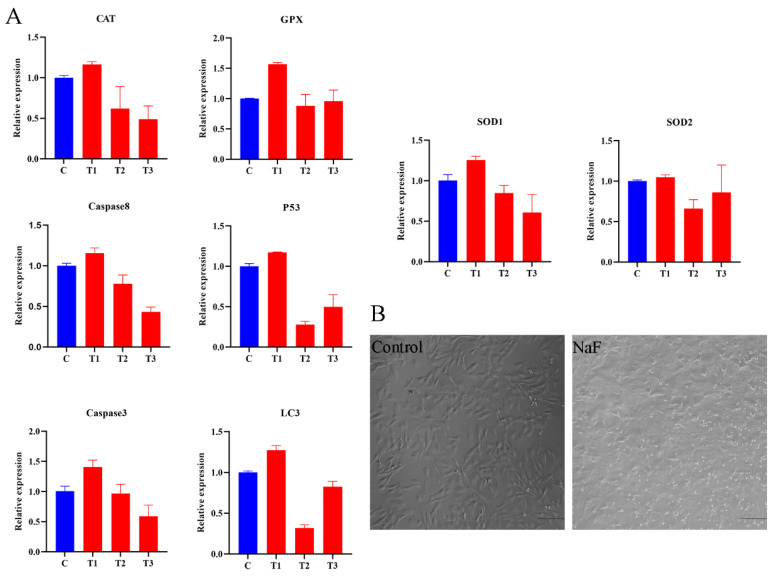
NaF final concentration screening. (**A**) qRT-PCR results of *CAT*, *GPX1*, *SOD1*, *SOD2*, *LC3*, *CASP3*, *CASP8*, and *P53* in control and experimental groups. (**B**) Cell morphology of control and experimental groups (10×, 100 µm).

According to the qRT-PCR results, the relative expressions of *GPX1*, *SOD2*, *LC3*, and *P53* showed an increase–decrease–increase pattern, reaching their lowest point in the 4 mM treatment (Figure 2A), indicative of a critical transition in expression. Conversely, the relative expression of *CAT*, *SOD1*, *CASP3*, and *CASP3* showed a tendency to increase and then decrease in a dose-dependent manner. The expression in the 4 mM-treated group was lower than that in the CK group. Based on the results of the CCK8 experiment, a final working concentration of 4 mM NaF was selected for RNA-Seq. The morphology of the ovine follicular GCs in the control and experimental groups is shown in Figure 2B.

### 3.3. Analysis of RNA-Seq Data and qRT-PCR Validation

**Figure 3:**
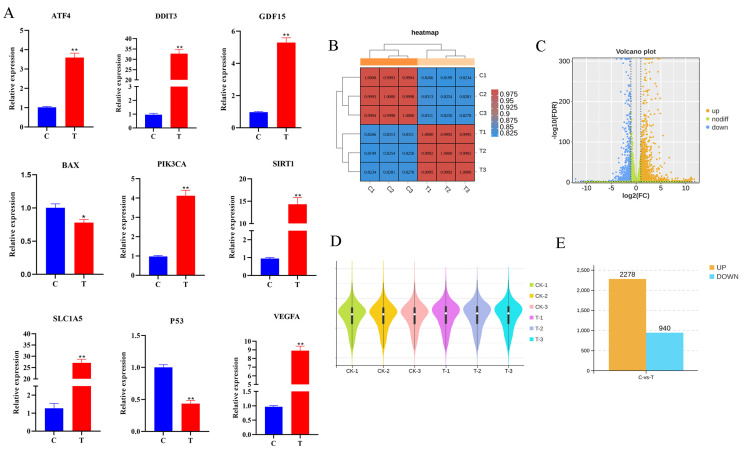
Analysis of RNA-Seq data and qRT-PCR validation. (**A**) qRT-PCR results of randomly selected *ATF4*, *DDIT3*, *GDF15*, *BAX*, *PIK3CA*, *SIRT1*, *SLC1A5*, *P53*, and *VEGFA* in control and experimental groups. (**B**) Heat map of sample. (**C**) Differential gene volcano plot. (**D**) Violin plot of samples. (**E**) Bar graph of differential genes. All experiments were repeated at least 3 times. * *p* < 0.05, ** *p* < 0.01.

Nine randomly selected genes (*ATF4*, *DDIT3*, *GDF15*, *BAX*, *PIK3CA*, *SIRT1*, *SLC1A5*, *P53*, and *VEGFA*) were subjected to qRT-PCR validation (Figure 3A). The consistency of the qPCR results with the RNA-Seq results indicates that the sequencing results are credible. The heat map of the sample relationships (Figure 3B) shows that the correlation coefficient between individual samples exceeded 0.820. The volcano plot (Figure 3C) shows the differential expression of the genes between the control and experimental groups. Genes closer to the ends of the horizontal axis indicate greater differences. Upregulated genes are indicated by yellow dots, downregulated genes are indicated by blue dots, and green dots indicate no significant difference. Additionally, the violin plot (Figure 3D) demonstrates the absence of significant outliers within the samples. The numbers of DEGs in each group that met the threshold screen were counted. The bar graph (Figure 3E) shows that of the 3218 differential genes screened, 2278 (yellow) were upregulated and 940 (blue) were downregulated.

**Figure 4:**
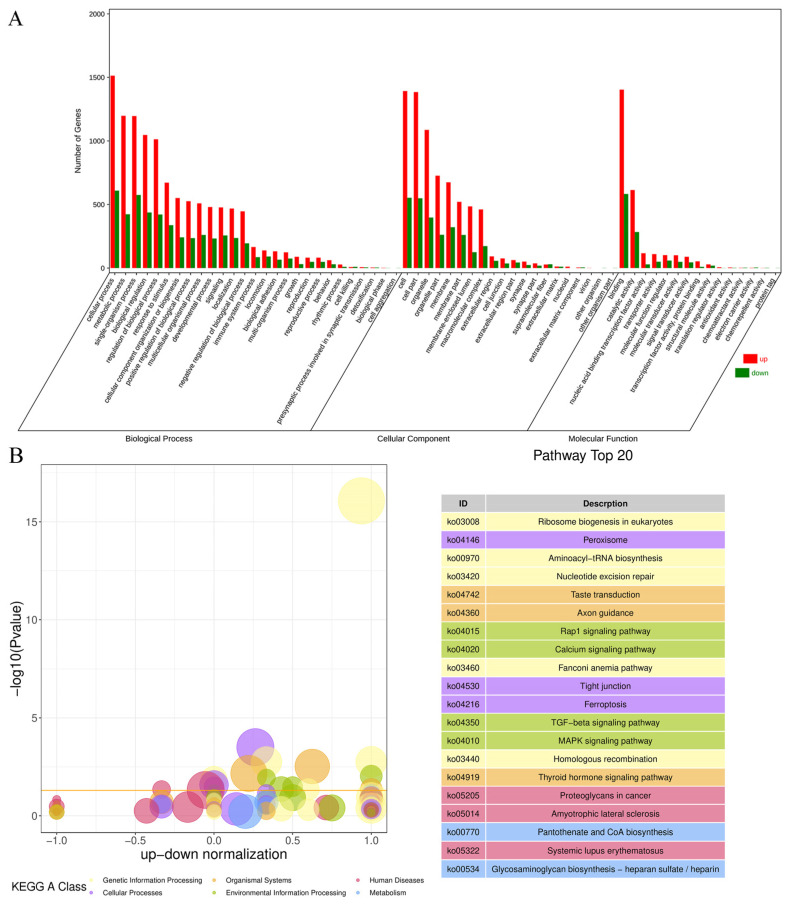
The results of GO and KEGG analysis. (**A**) GO enrichment classification histogram. (**B**) KEGG enrichment bubble diagram.

The results of the GO analysis (Figure 4A) reveal that the DEGs were categorized into 62 secondary entries involving three categories: biological processes, cellular components, and molecular functions. The largest proportions of the 27 secondary entries in the bioprocess classification were cellular, metabolic, and single-organism processes. The largest proportions of the 20 secondary entries in the cellular component classification were cells, cell parts, and organelles. The largest proportions of the 15 secondary entries in the molecular function classification were for binding, catalytic activity, and nucleic acid-binding transcription factor activity. The results of the KEGG analysis (Figure 4B) highlight that the impact of fluoride on ovine follicular GCs are primarily associated with genetic and environmental information processing and cellular processes. These pathways encompass nucleotide excision repair, ribosome biogenesis in eukaryotes, the calcium signaling pathway, TGF-beta signaling pathway, MAPK signaling pathway, peroxisome, tight junction, and ferroptosis.

**Figure 5:**
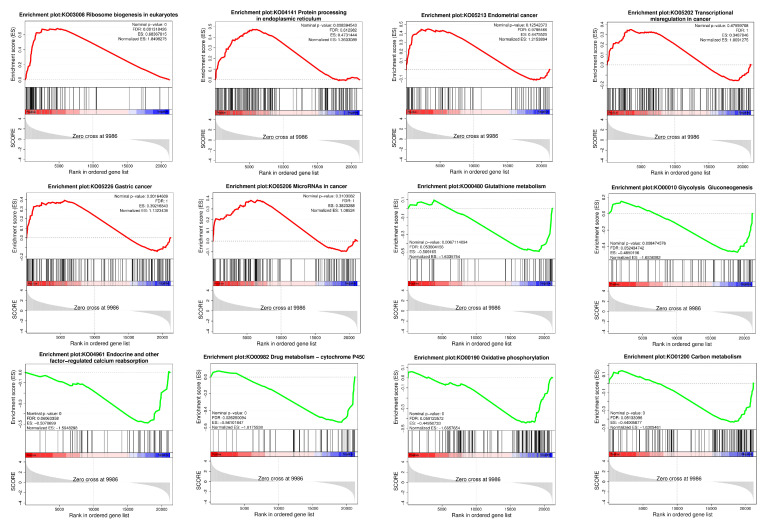
Results of GSEA. The red curve represents the activated pathway, and the green curve represents the inhibited pathway.

The GSEA of the sequencing results shows that pathways related to cancer and protein processing in the ER were activated, whereas pathways related to metabolism and endocrine and oxidative phosphorylation were inhibited (Figure 5).

**Figure 6:**
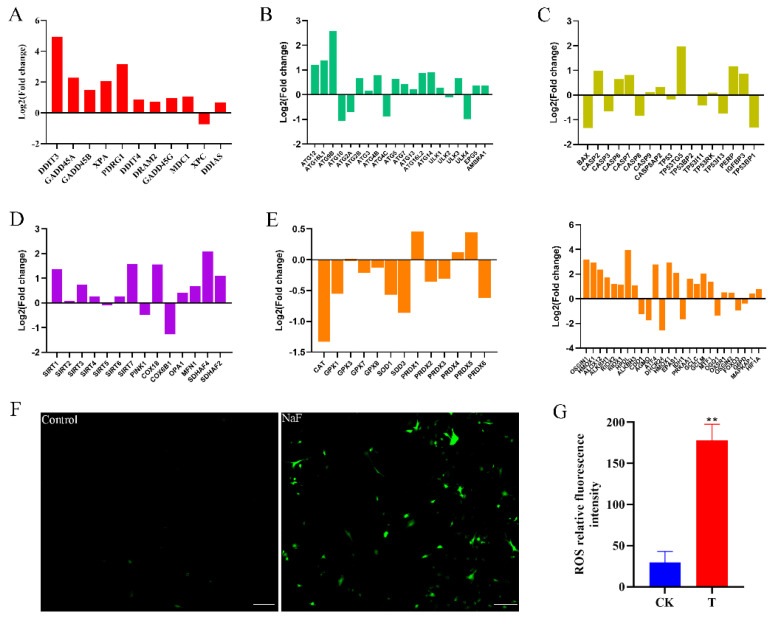
Histogram of RNA-Seq gene expression visualization. (**A**) Histogram of logarithmic values of DNA damage-related genes in RNA-Seq data. (**B**) Histogram of logarithmic values of autophagy-related genes in RNA-Seq data. (**C**) Histogram of logarithmic values of apoptosis genes in RNA-Seq data. (**D**) Histogram of logarithmic values of mitochondria-related genes in RNA-Seq data. (**E**) Histogram of logarithmic values of oxidative stress-related genes in RNA-Seq data. (**F**) Detection of ROS in control and experimental groups (10×, 100 µm). (**G**) ROS relative fluorescence intensity. ** *p* < 0.01.

Transcriptomic data screening identified the genes linked to cellular autophagy, oxidative stress, apoptosis, DNA damage, and mitochondria (Figure 6A–E). The bar graph results indicate that genes related to cellular autophagy, DNA damage, apoptosis, and oxidative stress were activated, whereas antioxidant-related gene expression was suppressed. The ROS assay was performed on control and fluoride-treated cells using an ROS assay kit, showing that the toxic effects of fluoride led to oxidative stress and intracellular accumulation of ROS (Figure 6F,G).

**Figure 7:**
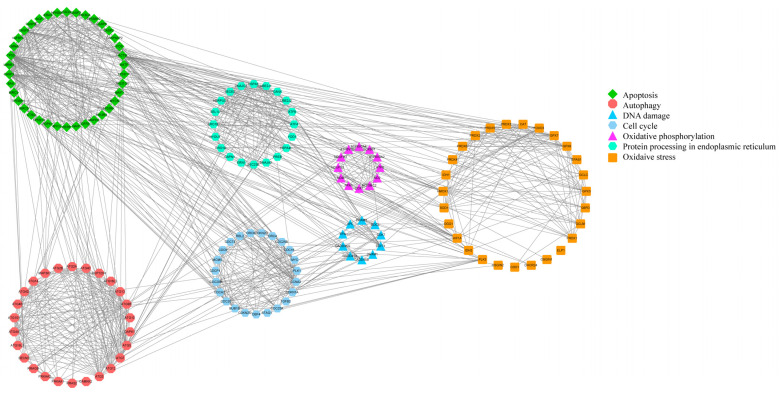
PPI network diagram of 156 DEGs.

**Figure 8:**
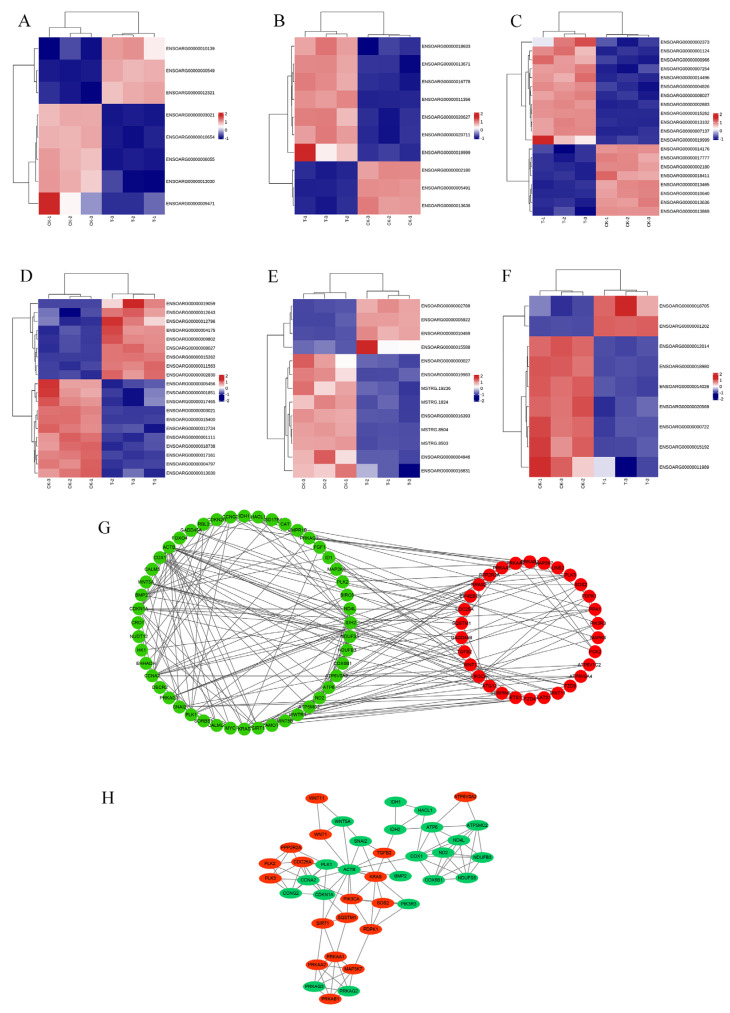
Results of DEG classification analysis of RNA-Seq. (**A**) Heat map of adherens junction-related genes. (**B**) Heat map of cancer-related genes. (**C**) Heat map of cellular senescence-related genes. (**D**) Heat map of Hippo pathway-related genes. (**E**) Heat map of oxidative phosphorylation-related genes. (**F**) Heat map of peroxisome-related genes. (**G**) PPI network map of DEGs. (**H**) proteins with ≥4 connections.

The PPI network diagram of 156 DEGs was constructed using STRING (Figure 7), and the 156 screened DEGs were categorized into seven classes: apoptosis, oxidative stress, autophagy, DNA damage, cell cycle, protein processing in the ER, and oxidative phosphorylation. The PPI network plot shows that the screened DEGs were closely associated with autophagy, oxidative stress, and apoptosis. Furthermore, 78 DEGs implicated in oxidative phosphorylation, endometrial cancer, adherens junctions, peroxisomes, and cellular senescence were categorized based on a literature review. The heat map (Figure 8A–F) shows that a large number of genes related to cancer and oxidative stress were activated. A PPI network map of the DEGs was constructed using STRING, and proteins with ≥ 4 connections were screened (Figure 8G,H).

### 3.4. Inference Diagram of the Mechanism of Fluoride on Ovine Follicular GCs

**Figure 9:**
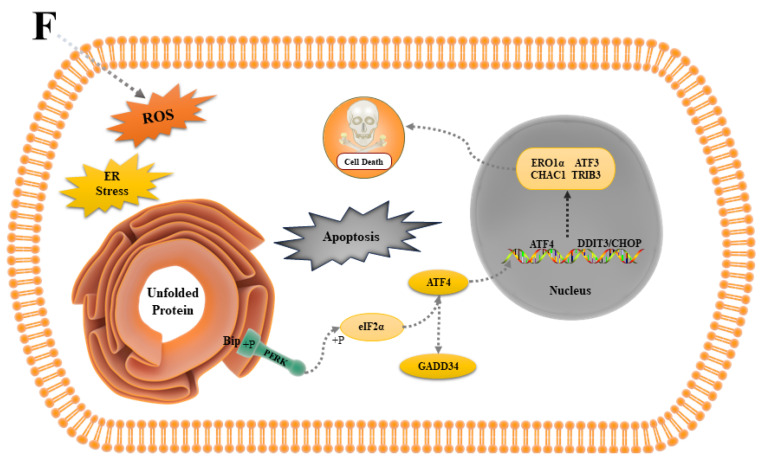
Diagram of the mechanism fluoride in ovine follicular GCs. (F: fluoride).

We reviewed the relevant literature and initially hypothesized that fluoride damages ovine follicular GCs by activating apoptosis-related genes, such as *CASP3*, *BAX*, and *BCL-2*. However, analysis of the histological results revealed that this was not the case and that cell death was not dependent on *Caspase* but closely related to ER stress. We constructed an extrapolated diagram of the possible mechanisms by which fluoride damages ovine follicular GCs (Figure 9).

## 4. Discussion

With the increasing frequency and scope of application of fluoride-containing compounds, research on fluoride exposure, hazards, and toxicological mechanisms has attracted considerable attention. GCs reflect the state of follicular development to a certain extent and are ideal cell models for studying cell proliferation, apoptosis, cell–cell interactions, and their signaling pathways. In this study, we investigated the toxic effects of fluoride on ovarian follicular GCs based on RNA-Seq.

Different concentrations of fluoride have varying effects on the biological characteristics and function of cells. The fluoride concentration examined in most in vivo studies is 2.6–5 mM [36,37,38], while in vitro studies (including cytotoxicity and genetic changes) suggest effective concentrations ranging from 0.1 to 5 mM [39,40,41]. The concentration of fluoride in human serum is usually maintained at a low level (0.01 mM), rarely exceeding 0.05 mM even in areas with high fluoride exposure [42]. In vitro fluoride concentrations are much higher than in normal serum levels due to differing cell susceptibilities. In in vitro experiments, fluoride acts directly on the cells, but the level of fluoride in vivo is affected by multiple factors and differs from the initial level after absorption and distribution. The CCK8 results show that the cell viability and fluoride concentration were linked in a dose-dependent manner, and the IC_50_ was 4.278. The majority of the cells died after being treated with 4.5 mM NaF, while 3.5 mM NaF did not induce any significant cellular variation. Based on IC_50_ (4 mM, 24 h) and previous studies, we chose 4 mM as the concentration for this study.

GO enrichment analysis of the DEGs showed that significantly enriched GO entries in the DEGs were mainly related to biological processes, including cellular processes, metabolic processes, single-organism processes, and biological adhesion. Conversely, KEGG enrichment analysis of the DEGs showed that fluoride is mainly related to genetic information processing, such as ribosome biogenesis in eukaryotes and nucleotide excision repair. In addition, it is related to cellular processes, such as tight junctions, peroxisomes, and ferroptosis, as well as environmental information processing, including the MAPK signaling pathway, TGF-beta signaling pathway, calcium signaling pathway, and human cancer.

The screening and analysis of transcriptomic DEGs revealed that the effects of fluoride on ovine follicular GCs were mainly related to cellular autophagy, the promotion of cellular oxidative stress, and the inhibition of cell cycle progression. Specifically, the expressions of *ATG12*, *ATG9B*, *ATG16L1*, *EPG5*, and *AMBRA1* indicated that fluoride induced autophagy in the ovine follicular GCs. Activation of the sirtuin family genes *SIRT1*, *SIRT2*, *SIRT3*, *SIRT4*, *SIRT6*, and *SIRT7* and oxidative stress-related genes *OSGIN1*, *HMOX1*, *ALOX12*, *RIOX1*, and *RIOX2* suggested that fluoride may lead to oxidative stress in ovine follicular GCs. The downregulation of the antioxidant genes *CAT*, *GPX1*, *SOD1*, and *SOD3*, and select genes from the PRDX family indicates that fluoride leads to a decrease in the antioxidant capacity of ovine follicular GCs, which ultimately leads to the accumulation of ROS.

Interestingly, we identified a close relationship between the effect of fluoride on ovine follicular GCs and ER stress during data mining and plotted the hypothesized process as a mechanism of action (Figure 9). We deduced that after fluoride entered the cell, it stimulated the cell to produce a stress response. Consequently, the ER internal environment was disturbed, resulting in the activation of PERK, which upregulated the expression of *ATF4*. *ATF4* then induced the expression of related factors, such as *DDIT3*/*CHOP* and *CHAC1*. Ultimately, the cells died because of overoxidation.

The synthesis, folding, and modification of proteins within the ER are tightly regulated, as they are fundamental to vital cellular functions and ultimately determine cell fate. The ER is very sensitive to stimuli from the internal and external environments, and under steady-state conditions, Bip proteins bind to ER stress sensors (three ER transmembrane proteins, ATF6, IRE1, and PERK) and remain inactivated [43]. When external environmental stimuli (e.g., hypoxia and drug toxicity) disrupt the internal environment of the ER, the oxidative environment in the ER lumen is also disrupted, and ER dysfunction occurs, resulting in the accumulation of unfolded proteins or misfolded proteins in the ER lumen, as well as the dysregulation of calcium homeostasis. In response, the ER stress response activates an adaptive effort to counteract these adverse factors and restore homeostasis in the body. When these countermeasures and severe imbalances persist, the ER stress response may initiate a pro-apoptotic program to eliminate defective cells and allow the entire organism to survive [44]. The above ER sensors are released, resulting in ER stress, which induces an unfolded protein response [45,46,47,48,49].

PERK, a member of the eIF2α protein kinase family, shares similarities with IRE1α. It is a type I membrane protein localized in the ER. The N-terminal region senses ER stress signals, featuring a non-ligand-dependent dimerization structural domain. During non-ER stress conditions, the dimerization site is concealed by Bip. The C-terminal region contains a functional serine/threonine protein kinase domain but lacks nucleic acid endonuclease activity. Specifically, the activation of PERK phosphorylates serine at position 51 of eIF2α, leading to the downregulation of overall intracellular protein synthesis. Phosphorylated eIF2α facilitates the translation of the *ATF4* transcription factor, which subsequently induces the expression of *CHOP*. *CHOP* activation promotes the expression of its key targets, *GADD34* and *CHOP*, thereby promoting the downstream expression of ER oxidoreductin 1 (*ERO1*), inducing an overoxidized state in the ER lumen and resulting in apoptosis. This process places the ER lumen in an overoxidized state, resulting in apoptosis [50,51].

This experiment was performed only on ovine GCs under only in vitro conditions. Owing to the limited life span of primary cells in culture, the growth potential was limited, which limited the feasibility of long-term experiments and caused objective errors in the experiments to some extent. However, isolated primary cells closely resemble in vivo conditions in animals, retain the specificity of their source tissues or organs, and have biological authenticity. They are ideal models for studying physiological and pathological processes and drug reactions. In the future, we plan to conduct animal experiments to further support our findings.

To date, the toxic effects of fluoride remain a major challenge in many parts of the world. Fluoride poisoning, through any route, has deleterious effects on sheep, causing serious economic losses and social impact. Therefore, prevention remains the best way to reduce toxicity in animals. In the future, we aim to validate the key factors in specific signaling pathways to confirm our conjecture and screen antagonistic drugs to mitigate the cellular damage caused by fluoride. However, further studies are needed to elucidate the mechanisms involved. This study offers a comprehensive database and theoretical framework for further exploration into the mechanisms underlying fluoride toxicity in follicular GCs. Additionally, it provides insights for screening antagonistic drugs, reducing livestock loss, and generating economic benefits.

## 5. Conclusions

In this study, we used RNA-Seq to investigate the mechanism underlying the effects of fluoride on ovine follicular GCs, hypothesizing that it acts through ER stress. Fluoride entry into the cell stimulates a stress response in the cell, activating the transcription factor *ATF4* in the PERK pathway, which induces *DDIT3*/*CHOP*, followed by the activation of other factors, such as *CHAC1*, *ATF3*, *ERO1α*, and *TRIB3*, ultimately resulting in cell death. These findings suggest that fluoride can cause DNA damage and cellular ROS accumulation in ovine follicular GCs.

## Figures and Tables

**Table 1:** Primer sequences for qRT-PCR assays.

Primers	Primer Sequences	NCBI Reference Sequence	Product Length (bp)
*CAT*	CCAGCCCTGACAAAATGCTT	XM_060400055.1	242
	AAAGCGGGTCCTATGTTCCA		
*SOD1*	GGCAATGTGAAGGCTGACAA	NM_001145185.2	130
	TGCCCAAGTCATCTGGTCTT		
*SOD2*	GGACAAATCTGAGCCCCAAC	NM_001280703.1	180
	CAATCTGTAAGCGTCCCTGC		
*GPX1*	CAGTTTGGGCATCAGGAAAAC	XM_004018462.5	100
	CGAAGAGCATGAAATTGGGC		
*CASP3*	ACGGAAGCAAATCAGTGGAC	XM_060406953.1	167
	GGTTTCCCTGAGGTTTGCTG		
*CASP8*	AGTGAGTTGCAGACATCCGA	XM_060410232.1	172
	AGGTCTTGTCCAAAGCCTCT		
*P53*	TCTTCAGATCCGTGGGCGTA	NM_001009403.1	158
	TTTTATGGCAGGAGGGAGAAGG		
*BAX*	TTCCGACGGCAACTTCAACT	XM_027978594.3	211
	CCATGTGGGTGTCCCAAAGT		
*LC3*	ACGCCTCTCAGGAGACTTTTG	XM_004014953.4	121
	ACCTCAGTTGGTAACATCCCT		
*PIK3CA*	GAGGAGCCCCGAGCATTTCT	NM_006218.4	134
	AAGTGGATGCCCCACAGTTC		
*ATF4*	AGGAGGATGCCCACTCAGAT	XM_012158819.3	172
	TCTCCAGGAGGGTCGTAAGG		
*DDIT3*	GCTGCCCTTCCCTTTTTGACTA	XM_060412904.1	111
	CTCAGTAAGCCAAGCCAGAGA		
*SIRT1*	GAGGCGGTTGAAAGATGGCG	XM_015104377.4	76
	TCAGCCGCCACTACCGAG		
*VEGFA*	ACCAAAGCCAGCACATAGGA	NM_001025370.3	105
	GCCTCGGCTTGTCACATTTTTC		
*GDF15*	TGCTCATGTTCTCCTGGCTG	XM_004008419.5	179
	GTCTTCCCAGGTCTGGTTCG		
*SLC1A5*	CTTGATCTTGGCCGTGGACT	XM_027978525.2	99
	TCCAGGTAACTCGGGAGGAG		

## Data Availability

The data that support the findings of this study are available from the corresponding author upon reasonable request.
